# Effect of Flavonoid-Coated Gold Nanoparticles on Bacterial Colonization in Mice Organs

**DOI:** 10.3390/nano10091769

**Published:** 2020-09-07

**Authors:** Sundus Riaz, Nosheen Fatima Rana, Irshad Hussain, Tahreem Tanweer, Afrah Nawaz, Farid Menaa, Hussnain A. Janjua, Tahseen Alam, Amna Batool, Ayesha Naeem, Maryam Hameed, Syed Mohsin Ali

**Affiliations:** 1 Department of Biomedical Engineering and Sciences, School of Mechanical & Manufacturing Engineering, National University of Sciences & Technology, Islamabad 44000, Pakistan; sundusriaz_fuuast@yahoo.com (S.R.); ttanveer.pg@smme.edu.pk (T.T.); anawaz.bmes16smme@student.nust.edu.pk (A.N.); abatool.phd20sme@student.nust.edu.pk (A.B.); anaeem.phd19bmessmme@student.nust.edu.pk (A.N.); maryam_hameed@outlook.com (M.H.);; 2 Ministry of National Food Security and Research, Pakistan Agricultural Research Council, Karachi 75270, Pakistan; 3 School of Science and Engineering, Lahore University of Management Sciences, Lahore 54000, Pakistan; ihussain@lums.edu.pk; 4 Department of Internal Medicine and Nanomedicine, California Innovations Corporation, San Diego, CA 92093, USA; dr.fmenaa@gmail.com; 5 Atta Ur Rahman School of Applied Biosciences, National University of Sciences & Technology, Islamabad 44000, Pakistan; principal-asab@nust.edu.pk (H.A.J.); tahseen260@gmail.com (T.A.)

**Keywords:** flavonoids, *Berberis lycium*, *Enterococcus faecalis*, drug delivery, infectious diseases

## Abstract

Multidrug resistance (MDR) has been a potentiator for the exploration of antibiotics. Nano drug delivery systems have opened new avenues to overcome this challenge. Although antibacterial nanocarriers are extensively realized, their effect on the bacteria residing inside the tissues and their toxicity is rarely explored. This study investigated the effects of flavonoid coated gold nanoparticles (FAuNPs) on the colonization of *Enterococcus faecalis* in the mouse liver and kidneys. Flavonoids were extracted from the leaves of *Berberis lycium* Royle and used to stabilize gold following a green synthesis approach. FAuNPs were characterized by ultraviolet-visible (UV-Vis) spectroscopy, Fourier-transform infrared spectroscopy (FTIR), scanning transmission electron microscopy (STEM), X-ray powder diffraction (XRD), and energy-dispersive X-ray spectroscopy (EDS). FAuNPs showed significantly higher reduction in bacterial counts in in-vitro and in-vivo in mice organs as compared to the free flavonoids owing to their biocompatibility and effectiveness.

## 1. Introduction

Functional nanoparticles (NPs) have a wide range of medical applications that encompasses a broad spectrum of fields, including imaging, molecular diagnosis, and targeted drug delivery [1,2]. As drug nanocarriers, they move inside the body to repair damaged tissues, cross the cell barriers, and access those cells and tissues where other drugs/antibodies cannot reach in appropriate concentrations [2]. Their superior features are due to high surface area-to-volume ratio, small size, stability, and biocompatibility [2,3].

The history of the gold nanoparticles (AuNPs) for the delivery of antibacterial agents dates to more than a decade. Various stable complexes of antibiotics with colloidal gold have been developed. These include gold complexes with vancomycin, ciprofloxacin, and fluorouracil [4,5,6]. However, ampicillin, kanamycin, streptomycin, and many other antibiotics form unstable complexes with gold [7,8,9]. Later, gold nanoparticles functionalized with ampicillin [10,11], vancomycin [12], and lysozyme [13] have been reported against various strains, such as *Pseudomonas aeruginosa*, *Enterobacter aerogenes,* and multidrug resistance (MDR) *Staphylococcus aureus*.

The hard bacterium, *Enterococcus faecalis,* is an opportunistic pathogen that can withstand high environmental stresses [14]. Despite being mammalian gut residents, it also causes nosocomial infections such as urinary tract infection (UTI), bacteremia, surgical wound infection, and endocarditis [15]. Its characteristic feature resides in its acquired and intrinsic resistance against main groups of antibiotics such as aminoglycosides, β-lactams, cephalosporins, glycopeptides, tetracyclines, and trimethoprim-sulfamethoxazole [16,17]. This MDR is manifested by mutations or by the horizontal exchange of foreign genetic material through the transfer of plasmids and transposons [18]. Antibiotic development against Enterococci is, therefore, an active and challenging research area.

Various nanocarrier systems were documented to be effective against *E. Faecalis*.These include gold nanorods for phototherapy [1], vancomycin bound biogenic AuNPs [19], calcium hydroxide NPs [20], chitosan NPs [21], Ag–Ca–Si mesoporous NPs [22], silver NPs [23], and polysaccharide-maghemite composite NPs [24]. However, these studies are focused on in-vitro antibacterial activity. NPs stability and activity under physiological conditions are also a matter of interest as many factors can alter the inherent antibacterial activity of NPs, such as pH and temperature [25]. Besides, plasma proteins and plasma components can also interact with the NPs surface, and this can affect their biodistribution [26,27,28], cellular uptake, and bioactivity [29,30,31]. Recently, a few in vivo studies have been carried out that include combined therapy of silver NPs, and visible blue light against *Pseudomonas* [32], quercetin loaded PLGA for *Escherichia coli* [33], and tridecaptin-antibiotic conjugates against *Klebsiella pneumoniae* [34].

AuNPs are not considered as antibacterial agents individually; however, when conjugated with small active substances (e.g., antibiotics and antibodies), they usually exhibit more potent antimicrobial activity [4,5,6]. Plant, bacterial, fungal, yeast and algal extracts were frequently used for the preparation of bioactive AuNPs according to previous studies. [35,36,37]. The bioactive compounds in these extracts not only reduce gold (Au^+3^) but also impart characteristic bioactive properties to these NPs [38]. Flavonoids are one of the extensively studied bioactive secondary metabolites from plants. The antibacterial activity of Flavonoids is ascribed to intracellular targeting such as inhibition of bacterial enzymes, damage to cytoplasmic membranes and cell wall components, inhibition of the bacterial efflux pump, and disruption of energy metabolism pathways [39]. Flavonoids can be extracted from *Berberis lycium* Royle, a spiny shrub prevalent in milder climates and subalpine regions.They are commonly used to treat a wide variety of human pathologies in the Pakistan, India, and Bangladesh Indian Himalayan Region. The phytochemical analysis of *B. Lycium* plant parts indicated the presence of important contents, including flavonoids, phenols, alkaloids, terpenoids, tannin, fat, and resin [40].

Moreover, its leaves and fruits contain a high amount of different nutritive components such as vitamin C, calcium, sulfur, protein, fiber, fat, palmitine, and berberine [40]. Importantly, *B. lycium* roots contain berberine, a quaternary ammonium salt from the protoberberine group of benzylisoquinoline alkaloids, which is reported for wide medicinal applications [40]. However, the bioactivities of leaves of this plant have not yet been investigated. Therefore, the present research focuses on the green synthesis of flavonoid-coated gold nanoparticles (FAuNPs) from methanol extracts of *B. lyceum,* its physical characterization, and the determination of its potential effect on colonization of *E. faecalis* both in vitro and in vivo. Herein, the biosynthesized AuNPs exhibit special antibacterial properties due to their stabilization by flavonoids. To the best of our knowledge, this is the first in-vivo study that investigates the antibacterial activity of NPs against Gram-positive bacterial colonization in mice organs such as liver and kidneys.

## 2. Materials and Methods

*B. lycium* leaves were sampled from Patriata, Murree, Pakistan. Chemicals were purchased from Sigma-Aldrich (St Louis, MO, USA), except where stated. Tetrachloroauric acid trihydrate (HAuCl_4_·3H_2_O) was purchased from Merk (Münch, Germany). All protocols employed were approved by the Internal Review Board (IRB), Department of Biomedical Engineering and Sciences, School of Mechanical and Manufacturing Engineering, National University of Sciences and Technology (NUST).

### 2.1. Bacterial Strains

Gram-positive bacterial strains used in the study included *E. faecalis* JH2-2 (derived from the parental strain JH2) [41], *Bacillus cereus* (soil isolates), and *S. aureus* (ATCC 6538). Gram-negative strains included *P. aeruginosa* (ATCC 9027), *Salmonella Typhi* (ATCC 6539*),* and *E. coli* (ATCC 8739).

### 2.2. Flavonoid Extraction

Flavonoids were extracted using Soxhlet extractor (Sigma-Aldrich, St Louis, MO, USA) from *B. lycium* leaves in 80% methanol for 24 h. Methanol was subsequently evaporated by vacuum. The aqueous fraction was extracted with petroleum ether (40–60 °C), ethyl acetate, and diethyl ether [42] (Figure S1). Free flavonoids (FF) are aglycones, while in naturally occurring conjugated flavonoids (CF), most commonly as glycosylated and methylated derivatives, the fatty acid carbon chain is linked onto the primary –OH group on the glucose moiety of the flavonoids. Flavonoids screening was performed using Shinoda’s Test optimized by Mir et al. (2013) and Inalegwu and Sodipo (2013) [43,44]. FF and CF were dried using a rotary evaporator (Buchi Rotavapor R-200 system, Marshal Scientific, Cambridge, US).

### 2.3. Green Synthesis of FAuNPs

The solution of CF concentrated to 1 mg/mL in distilled water was dropwise added to 1 mM tetrachloroauric acid (HAuCl_4_). Optimization was rendered employing varying Flavonoid / HAuCl4 formulations ratios (1:1, 1:2, 1:3, 1:4, and 1:5). These formulations were kept at different varying (25, 40, 50, 60, 70, 80, and 100 °C) with constant magnetic stirring for 4 h. The pH of the formulations was adjusted to 4, 6, 8, and 10 using strong HCl and NaOH.

NPs were collected by centrifugation at 11,000× *g* for 10 min. FAuNPs were lyophilized by means of a freeze dryer (EYELA FDU-1000, Tokyo, Japan) for 24 h at 15 Pa. The concentration of AuNPs was evaluated with the help of Beer’s Law, i.e.,:

*A* = *E* × *I* × *C* (1)

where, *A* is the absorbance, *E* (M^−1^ cm^−1^) is the molar extinction coefficient, *I* is the path length (cm), and *C* is the concentration.

The UV-vis absorption spectra were recorded in the range of 300–800 nm. The extinction coefficient was obtained from the standard curve.

### 2.4. Physical Characterizations of FAuNPs

The FAuNPs synthesized in the study were characterized by UV-Vis spectroscopy, FTIR spectroscopy, XRD, and scanning transmission electron microscopy (STEM). Spectrophotometric analyses were carried out by UV-2800 BMS Scientific Technical Corporation (PVT) Ltd. Spectrophotometer (Shanghai, China). For FTIR analysis, the Perkin Elmer spectrum 100 instruments (Waltham, MA, USA) were used, and the spectra were recorded in the range of 4000–400 cm*^−^*^1^. XRD of FAuNPs was done using the scanning mode on WinX’POW. The X-ray diffractometer system (STOE: theta/theta, Darmstadt, Germany) functioned at 40 kV and a current of 30 mA with Cu K*α* radiation (l¼ 1.54064°A). STEM/EDAX (energy-dispersive analysis of X-rays) of FAuNPs was performed with a FEI NOVA, NanoSEM 450 (Hillsboro, Oregon, US) equipped with a STEM detector operated at 120 keV. STEM images of FAuNPs were then managed through Image-J software 1.8.0_112, windows (64 bit) version (NIH and LOCI, Madison, WI, USA) for their histogram analysis.

### 2.5. In-Vitro Stability of FAuNPs

FAuNPs were centrifuged at 10,000× *g* for a period of 10 min; the subsequent pellets were then resuspended in 2, 3, and 4 M NaCl solution and set aside at 37 °C for 24 h [45]. The impact of heat on FAuNPs was determined by heating 10 mL of FAuNPs at 100 °C for 30 min.

### 2.6. Antibacterial Susceptibility of Flavonoids by Qualitative Method

The antibacterial activity of FF, CF and crude extracts (CE) against various bacteria was performed using the Kirby–Bauer method. [46]. Disks were loaded with 10 μL of FF, CF, and CE separately against different Gram-positive (*E. faecalis, B. cereus,* and *S. aureus*) and Gram-negative bacteria (*P. aeruginosa, S. typhi,* and *E. coli*). The respective inhibition zone (ZI) was measured after incubation at 37 °C for 24 h.

### 2.7. Minimum Inhibitory Concentration (MIC) and Minimum Bactericidal Concentration (MBC) of CF and FAuNPs Against *E. faecalis*

The MIC of CF and FAuNPs against *E. faecalis* was determined using a standard broth microdilution method after 24 h of incubation at 37 °C with an inoculum of approximately 10^6^ CFU/mL. Column 1 functioned as a negative control which constituted only the medium, whereas column 2 functioned as a positive control, i.e., medium-plus bacterial inoculum. The MBC test was performed via inoculation of MIC broth on culture plates containing nutrient agar. The lowest concentration that showed less than 50% of visible bacterial growth after 24 h was taken as MBC.

### 2.8. Hemolysis Assay

The hemolytic activity was estimated according to the method described by Muhammad et al. (2016) [47]. Triton X-100 (0.5%) (Sigma Aldrich, St Louis, MO, USA) was used as a positive control. The absorbance was measured using a UV-2800 BMS Scientific Technical Corporation (PVT) Ltd. Spectrophotometer (Shanghai, China) at 550 nm.

### 2.9. Colonization of *E. faecalis* in BALB/c Mice

BALB/c mice (*n* = 30) were purchased from the National Institute of Health (NIH), Islamabad. Eight weeks-old BALB/c female mice (25–30 g) were kept under temperature 25 ± 2 *°*C and provided with a natural light (10 h) and dark cycle (14 h). Autoclaved tap water and a standard diet ad libitum were given to mice.

A well-established intravenous infection model was used for bacterial colonization in mice tissues [14,15,16]. Preculture was prepared in GM17 broth at 37 °C [14]. Brain heart infusion medium augmented with 40% filter-sterilized serum was injected with 100 µL of preculture and were incubated at the temperature of 37 °C with shaking while waiting for the OD_600_ to reach at 0.8. Cultures were centrifuged, and the subsequent pellets were washed with phosphate buffer saline (PBS) 1 M (composed of NaCl, KCl, Na_2_HPO_4_, and KH_2_PO_4_, accustomed to pH 7.4 with HCl). It is then suspended in sterile PBS. Bacterial suspensions of 100 µL (1 × 10^9^ cells/mL) were injected intravenously (tail vein) in each of the twenty-five female mice. The remaining five mice were untreated and thus served as negative controls.

### 2.10. In Vivo Antibacterial Activity of CF and FAuNPs

To assess the in-vivo antibacterial activity, both CF and FAuNPs were dissolved in PBS. Two infected groups (5 mice each) were treated with CF (5 mg/kg and 10 mg/kg), and the other two groups were treated with FAuNPs (400 µg/kg and 5 mg/kg). CF and FAuNPs were deliveredin the tail vein once a day for eight days starting from the seventh day of infection until the day of the challenge.

### 2.11. Statistical Analysis

Statistical analysis such as the average, standard deviation, and multiple group comparison analysis by using a one-way ANOVA was calculated by Graph pad prism (Graphpad, San Diego, CA, USA).

## 3. Results and Discussion

It is well assumed that NPs carrying antibiotics are found to be quite effective against resistant bacteria [48]. This is particularly due to the striking features of NPs attributed to their size and unique physiochemical properties at the nanoscale that help them to evade drug efflux pumps. Flavonoids, albeit depending upon their type and structure, often display poor absorption and bioavailability [49]. One way to combat the above constraints is to use drug nanocarriers [2,41]. For their development, flavonoids can act as reducing as well as a capping agent for the synthesis of metallic NPs. Green synthesis is, therefore, a better option than using toxic reducing agents, especially for biomedical applications.

Flavonoids were used for a range of medicinal applications, such as antibacterial, antiviral, antioxidative, anti-inflammatory, anticancer, cardio-protective, skin-protective, and antidiabetic activities [50]. Flavonoids can be obtained from *B. lycium*, which is a known medicinal plant containing a variety of bioactive constituents [40]. Medicinal plants are a good source of potent and safe natural extracts that could act as adjuvants or even be an alternative to costly antibiotics against which microbes are becoming resistant day by day. Moreover, these extracts are cost-effective and work effectively against a variety of microbes, including bacteria, fungus, and viruses [51,52,53].

### 3.1. Antimicrobial Activity of Flavonoids

**Figure 1:**
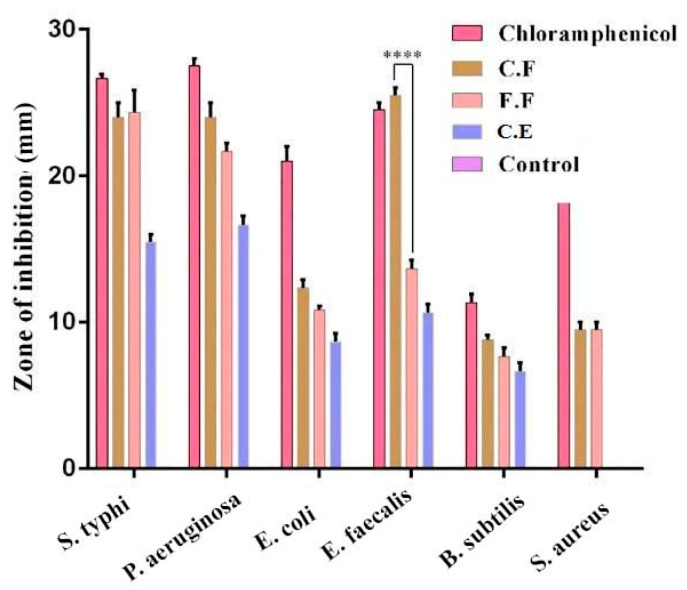
Antimicrobial activity (zone of inhibition; 500 µg/disc) of free (FF) and conjugated flavonoids (CF) and crude extracts (CE) against Gram-negative and Gram-positive bacteria. **** *p* ≤ 0.0001.

Since *B. lycium* leaves and their flavonoids have not been investigated before, we initially tested flavonoids against different Gram-positive (*B. cereus, S. aureus,* and *E. faecalis*) and Gram-negative bacteria (*P. aeruginosa, S. typhi,* and *E. coli*). Chloramphenicol and DMSO were utilized as positive and negative controls, correspondingly. Antimicrobial activity (ZI; 500 µg/disc) of FF, CF, and CE are presented in Figure 1. Both FF and CF showed maximum inhibition against Gram-negative bacteria, compared to CE. Among Gram-positive bacteria, significantly higher inhibition (*p* < 0.0001) against *E. faecalis* was observed for CF compared to that of FF. No statistically significant difference (*p* > 0.05) was noticed amongst the CF and chloramphenicol for this activity. Therefore, CF was used for the further steps of this study. Additionally, this research supports that the mechanism of action of flavonoids is well dependent on the type of flavonoids [49,54].

### 3.2. Synthesis and Physical Characterizations of FAuNPs

The green synthesized FAuNPs used in the study were characterized and analyzed by UV-Vis spectroscopy. FTIR spectroscopy, XRD, STEM, and EDS analysis (EDXA).

#### 3.2.1. Impact of Physico-Chemical Parameters on FAuNPs Synthesis and Its Stability

**Figure 2:**
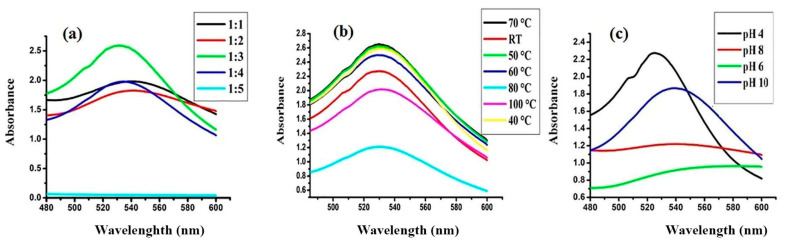
UV-Vis spectra of flavonoid coated gold nanoparticles (FAuNPs) synthesis at different (**a**) flavonoid/HAuCl_4_ ratios, (**b**) temperature, and (**c**) pH.

As shown in Figure 2a, variation in the *λmax* was observed when the different concentrations of HAuCl_4_ salt were utilized for the synthesis of FAuNPs. The absorption intensity gradually increased at 1:3 (Au:flavonoid), indicating the complete reduction of gold ions (Au^+^). The impact of temperature and pH on the formation of FAuNPs are presented in Figure 2b,c, respectively. Maximum absorbance was obtained for pH 4 and a temperature of 70 °C. Thus, these parameters were considered as optimal conditions for the formation of FAuNPs.

Concentration of FAuNPs in solution was determined using Beer’s law. The UV-Vis spectra showed maximum absorbance (2.6) at 529 nm. With 2.6 absorbance, 3 × 10^9^ M^−1^ cm^−1^ and 1 cm path length, the concentration of nanoparticles was calculated to be 8.6 × 10^−10^ M. The calculated concentration may have some inaccuracy owing to the polydispersity of the synthesized NPs. The calculated concentration is presented under the assumption that the prepared NPs are monodispersed.

**Figure 3:**
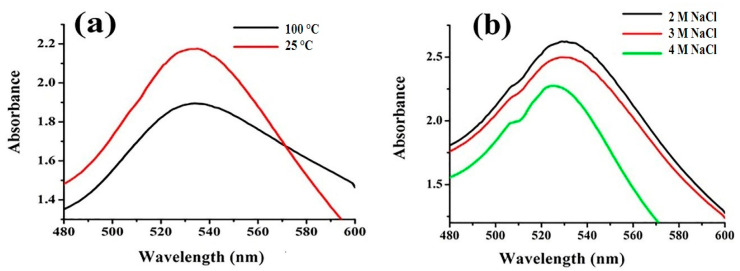
Effect of (**a**) temperature and (**b**) salt concentration on the stability of FAuNPs.

As shown in Figure 3a, the effect of temperature on the surface plasmon resonance (SPR) peak of FAuNPs allowed us to conclude that the effect of temperature on FAuNPs is negligible, has a minute reduction in absorbance while the surface plasmon peak do not shifted, and no aggregation observed.

Figure 3b shows the effect of varying concentrations of NaCl (2–4 M) on the SPR peak of FAuNPs. No effect on FAuNPs was noted by increasing the concentration of salt from 2 to 4 M NaCl solution even after a few weeks. It was observed that a higher concentration of salt increased the full width at half maximum (FWHM), or *λmax* also decreased, which results in reducednanoparticles stability. This decrease in *λ*max may be attributed to the aggregation of nanoparticles which was increased by Cl^−1^ ions. From these findings, it is inferred that gold nanoparticles are much more stable in water for long-term stability than in NaCl solution.

#### 3.2.2. FTIR Analysis of CF and FAuNPs

**Figure 4:**
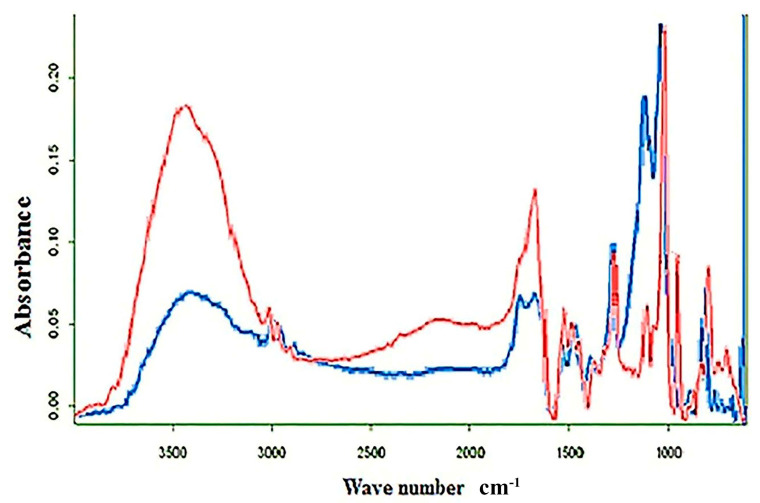
FTIR spectra of CF (red) and FAuNPs (blue).

FTIR spectroscopy data validated the conjugation of flavonoids with AuNPs since typically observed absorbance bands associated to flavonoids were observed in the area of 500–3500 cm*^−^*^1^ (Figure 4). Absorbance band at 3398 cm*^−^*^1^ can be ascribed to O-H stretching, 2960–2850 cm*^−^*^1^ to C–H stretching (CH_2_*,* CH_3_), 1652 cm*^−^*^1^ to C=O stretching, 1600 cm*^−^*^1^ to C=C stretching (aromatic), 1554 cm*^−^*^1^ to C–C stretching (aromatic), 1300 cm*^−^*^1^ to C–O stretching (ether linkage), 1210 cm*^−^*^1^ to C–O stretching (polyols), and 1100 cm*^−^*^1^ to C–OH stretching, while absorbance bands at 909 cm*^−^*^1^ and 850 cm*^−^*^1^ were both assigned to C–H bending vibrations out of plane [41,48].

#### 3.2.3. XRD Analysis of FAuNPs

**Figure 5:**
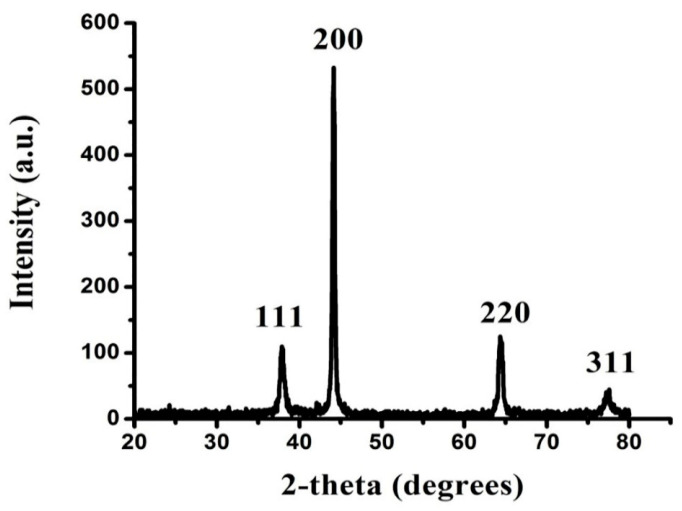
XRD analysis of FAuNPs.

The crystal structure of the biosynthesized FAuNPs was investigated by XRD. The Bragg reflection suggested that AuNPs were specifically indexed to a face-centered cube (FCC) crystal structure (Figure 5). In the XRD spectra of FAuNPs, peaks were achieved at 2θ values of 37°, 44°, 64° and 77° pertaining to the FCC gold reflections of 111, 200, 220 and 311, respectively. This pattern is perfectly suited to JCPDS card number 00-002-1095 [20]. The high intense peak (200) of FCC gold was detected in the sample. Peaks intensity exhibited a high degree of FAuNP crystal structure. The broad peaks of diffraction are attributable to the small size of the crystal.

From the Scherrer equation, the mean crystallite size of the FAuNPs was calculated [55].

(2) $$B=\frac{0.93\lambda }{\beta cos\theta }$$

where *λ* is the wavelength of the X-rays incident (*λ* = 1.54060 Å), β is the full width of the 200 diffractions half maximum and θ is the diffraction angle. The FAuNPs had a diameter of 37.6 nm.

#### 3.2.4. STEM Analysis of FAuNPs

**Figure 6:**
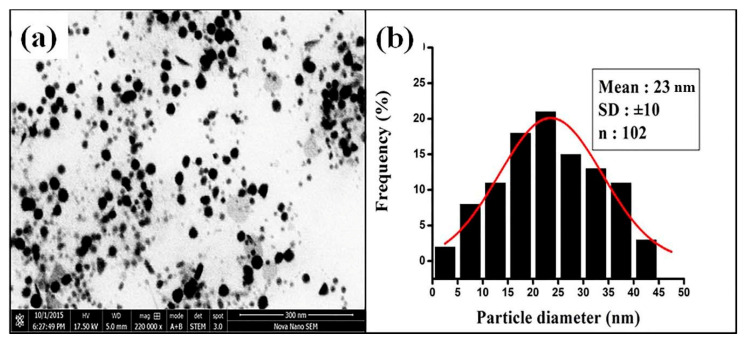
(**a**) Scanning transmission electron microscopy (STEM) image of FAuNPs and (**b**) particle size distribution of FAuNPs.

The size, shape and morphology of FAuNPs were analyzed using STEM (Figure 6). The FAuNPs comprised of spherical NPs (Figure 6a) measuring 3 to 50 nm in size (Figure 6b). Particle size distribution calculated from STEM image fitted with the Gaussian function indicating an average size of NPs of 23 nm (Figure 6b).

#### 3.2.5. EDS Analysis (EDAX) of FAuNPs

**Figure 7:**
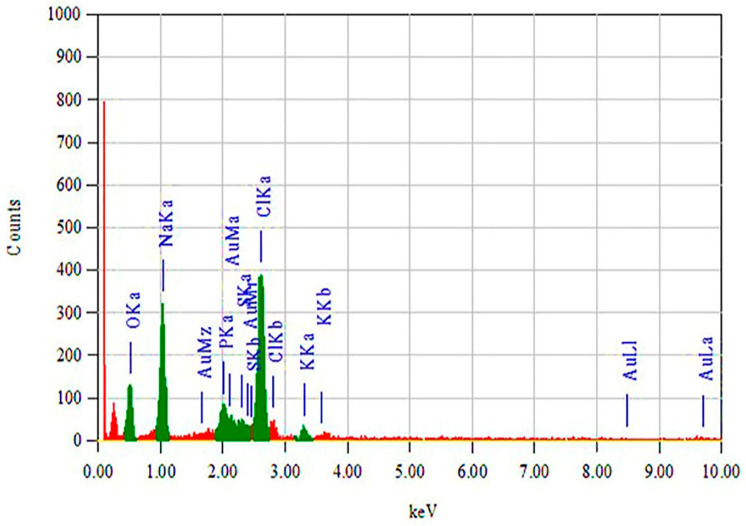
EDS analysis (EDXA) of FAuNPs.

The elemental composition of FAuNPs was studied by EDXA. Figure 7 showed the presence of Au at 4.42 wt %.

### 3.3. In Vitro Antibacterial Activity of FAuNPs

Enterococci are microorganisms with exceptional abilities, allowing them to survive in harsh environments. Thereby, the intrinsic and acquired ruggedness enable *E. faecalis* among *Enterococcus* sp. to be considered as the most persisting nosocomial causing pathogen [18]. In addition to this, reduced bactericidal concentration and activity of antibiotics to the site of the infection is also the culprit for radical-induced mutagenesis, a further supporter for MDR [56].

The disk diffusion test was conducted as an initial study to screen the antibacterial activity of flavonoids. Further investigation was done to determine the MIC and MBC values of CF and FAuNPs. The lowest concentration of an antimicrobial to inhibit the growth of the bacteria was considered as MIC, while the lowest concentration that allowed no growth after subculturing from MIC was regarded as MBC. This study showed that MIC for CF and FAuNPs against *E. faecalis* was 500 µg/mL and 25 µg/mL, respectively. The MBC of CF was about 2-fold higher than the final MIC (1.75 mg/mL), whereas the MBC for FAuNPs against *E. faecalis* was quite similar to their MIC (i.e., 25 µg/mL). At this concentration, less than 50% of bacterial growth was observed, indicating the potential of FAuNPs as a decent antibacterial agent at much lesser concentrations.

### 3.4. Hemolysis Assay

**Figure 8:**
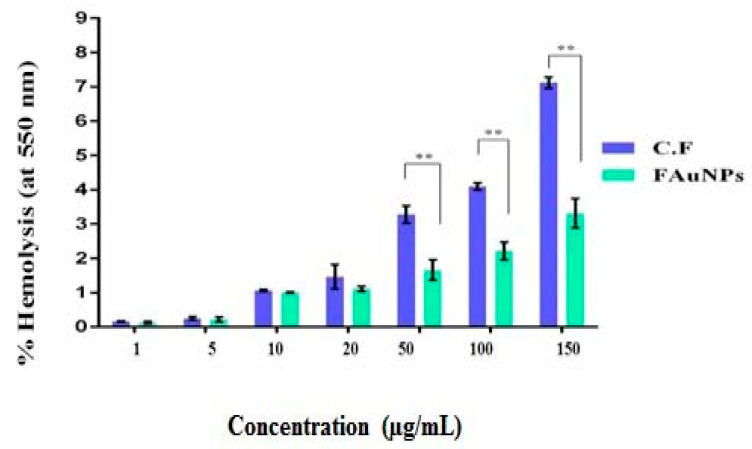
Hemolytic activity of CF and FAuNPs. ** *p* < 0.01.

Hemolysis is the breakdown of red blood cells (RBCs) and the discharge of their contents into the environment. So, when FAuNPs first enter the blood, they get in contact with RBCs [57]. To assess the impact of FAuNPs on RBCs, the hemolysis assay was performed to measure the hemolytic rate (%) when various concentrations (range: 0–150 μg/mL) of CF or FAuNPs were used. Hemolysis assay indicated that FAuNPs were more hemocompatible compared to CF, which the effect was seen as highly significant (** *p* < 0.01) when concentrations were used over 50 μg/mL (Figure 8). Hemolytic behavior of FAuNPs at different concentrations remained less than 5%, and according to ISO/TR 7406, this concentration is declared as safe.

### 3.5. In Vivo Anticolonizing Potential of CF and FAuNPs in Infectious Mice Model

**Figure 9:**
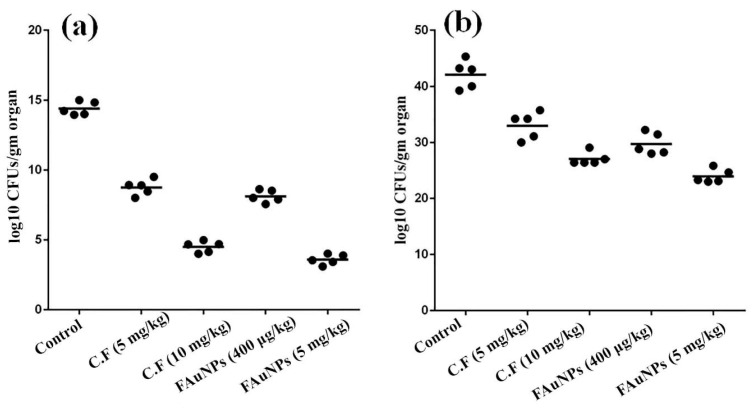
Effect of CF and FAuNPs, at respective MIC, on the colonization of *E. faecalis* in the kidneys (**a**) and liver (**b**) of mice.

*E. faecalis* infection model was prepared by intravenous (IV) administration of bacterial cells to study the anticolonizing activity of FAuNPs. The respective MIC dose of CF (i.e., 500 µg/mL) and FAuNPs (i.e., 25 µg/mL) was administered for seven days after infection in mice. Our results showed that diseased mice have reduced food intake and greenish feces. These symptoms were greatly improved on the 4th day of treatment with CF and the 2nd day with FAuNPs. IV administration of CF showed increased palpitation and itching in mice. This was, however, not the case with FAuNPs treatment. After a week of treatment, all mice were sacrificed, and their liver and kidneys were removed to measure the viable bacterial count. Figure 9 a,b show bacterial counts (log_10_ CFU/gm of the organ) in infected kidneys and liver, respectively.

The in-vivo results showed that the mice treated with FAuNPs have significantly reduced bacterial colonization in the liver (*p* = 0.001) and kidneys (*p* = 0.001) when compared to that of C.F-treated mice. No effect was found on the size, texture, and weight of the liver and kidneys by CF and FAuNPs-treated mice as compared to the normal mice. To the best of our knowledge, this is the first study that investigated the in-vivo activity of flavonoids against bacterial colonization in mice.

## 4. Conclusions and Perspectives

The enhanced antibacterial activity of bioactive compounds in nanoformulations has been extensively studied. However, most of such studies are focused on in-vitro aspects. The present study was focused on a green synthesis and characterizations of FAuNPs, which were further assessed for their in-vitro and in-vivo antibacterial potential against Gram-positive bacterium *E. faecalis*. The FAuNPs were successfully synthesized, and different physical characterizations confirmed their formation. The synthesized FAuNPs were sphere-shaped shaped with a 23 nm diameter average size. The results for optimizations revealed that a 1:3 ratio of Au and flavonoid, a pH of 4, and a temperature of 70 °C are effective conditions for FAuNPs formation. Stability results showed that temperature had negligible effect on stability of optimized FAuNPs. However, FAuNPs cannot remain stable in salt solution. These FAuNPs when tested for antibacterial activity showed enhanced activity against *E. faecalis* with MIC of 25 µg/mL. Similarly, they also showed reduced bacterial colonizing activity of *E. faecalis* in liver and kidneys of the mice. FAuNPs were more biocompatible owing to its reduced hemolytic behavior with varying concentrations. The results of this study conclude that FAuNPs can be very effective antibacterial agents against *E. faecalis* infections. The present study was based on a non-biofilm forming *E. faecalis* strain. In the future, further studies are required for biofilm-forming and vancomycin-resistant *E. faecalis* strains.

## Supplementary Materials

The following are available online at https://www.mdpi.com/2079-4991/10/9/1769/s1, Figure S1: Extraction procedure for free and conjugated flavonoids.

## Abbreviations

*B. lyceum:*	*Berberis lycium*
C.E:	Crude extract
C.F:	Conjugated flavonoids
*E. faecalis:*	*Enterococcus faecalis*
EDS:	Energy-dispersive X-ray spectroscopy
FAuNPs:	Flavonoid gold nanoparticles
F.F:	Free flavonoids
FTIR:	Fourier-transform infrared spectroscopy
HAuCl_4_:	Tetrachloroauric acid
IV:	Intravenous
MDR:	Multi-drug resistance
NPs:	Nanoparticles
RBCs:	Red blood cells
STEM:	Scanning transmission electron microscopy
UTI:	Urinary tract infection
UV-Vis:	Ultraviolet-visible
