# Supplementary material for: Effect of Flavonoid-Coated Gold Nanoparticles on Bacterial Colonization in Mice Organs
Source: Nanomaterials (Basel). 2020 Sep 7;10(9):1769. doi: 10.3390/nano10091769 (PMC7559771; doi:10.3390/nano10091769)
Supplement: Supplementary file 1 [file nanomaterials-10-01769-s001.pdf]

nanomaterials

Supplementary Material

Effect of Flavonoid-Coated Gold Nanoparticles on Bacterial Colonization in Mice Organs

Sundus Riaz [1,2], Nosheen Fatima Rana [1,*], Irshad Hussain [3], Tahreem Tanweer [1], Afrah Nawaz [1], Farid Menaa [4], Hussnain A. Janjua [5], Tahseen Alam [5], Amna Batool [1], Ayesha Naeem [1], Maryam Hameed [1] and Syed Mohsin Ali [1]

1 Department of Biomedical Engineering and Sciences, School of Mechanical & Manufacturing Engineering, National University of Sciences & Technology, Islamabad 44000, Pakistan; sundusriaz_fuuast@yahoo.com (S.R.); ttanveer.pg@smme.edu.pk (T.T.); anawaz.bmes16smme@student.nust.edu.pk(A.N.); abatool.phd20sme@student.nust.edu.pk (A.B.); anaeem.phd19bmessmme@student.nust.edu.pk (A.N.); maryam_hameed@outlook.com (M.H.); mehvishriaz4@gmail.com (M.R); sali.bmes17smme@student.nust.edu.pk(S.M.A.)

2 Ministry of National Food Security and Research, Pakistan Agricultural Research Council, Karachi, 75270 Pakistan

3 School of Science and Engineering, Lahore University of Management Sciences, Lahore 54000, Pakistan; ihussain@lums.edu.pk

4 Department of Internal Medicine and Nanomedicine, California Innovations Corporation, San Diego, CA, 92093 USA; dr.fmenaa@gmail.com

5 Atta Ur Rahman School of Applied Biosciences, National University of Sciences & Technology, Islamabad 44000, Pakistan principal-asab@nust.edu.pk (H.A.J.), tahseen260@gmail.com (T.A.)

* Correspondence: nosheen.fatima@smme.nust.edu.pk; Fax: +92-9085-6001

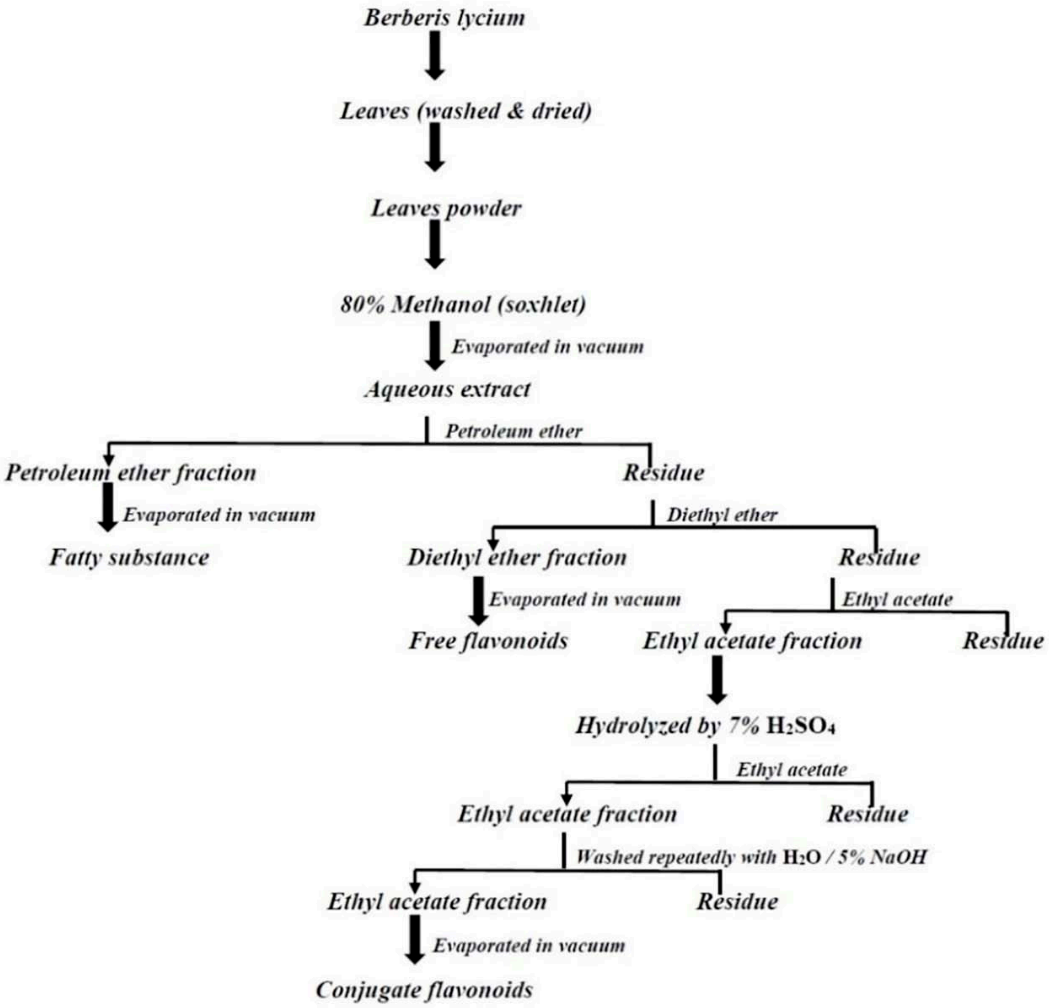

Figure S1. Extraction procedure for free and conjugated flavonoids.

© 2020 by the authors. Licensee MDPI, Basel, Switzerland. This article is an open access article distributed under the terms and conditions of the Creative Commons Attribution (CC BY) license (http://creativecommons.org/licenses/by/4.0/).
